# THE SOCIAL CONSTRUCTION OF RETIREMENT AND GENDER DIFFERENCES IN RETIREMENT TIMING

**DOI:** 10.1093/geroni/igz038.1405

**Published:** 2019-11-08

**Authors:** Michelle Silver

**Affiliations:** University of Toronto, Toronto, Ontario, Canada

## Abstract

Retirement is an ever-evolving, dynamic, and complex social construct we associate with the end of one’s career. For some the term is a bad word and a term that needs to be retired, while others can’t wait to retire and enjoy the good life. This paper examines a brief history of retirement and theoretical work from feminist gerontology, while focusing on gender differences in the social construction of retirement and policy implications of 10 different government pension plans. In doing so, it looks at policy implications associated with the standard retirement age tied to public pension plans in the United States, Canada, and the European Union. Findings indicate that women live longer than men in each country, yet women retire earlier and receive lower pensions than men. As the landscape surrounding women’s work experiences changes and concerns about gender equity in salaries and workplace compensation continue to be raised, this paper extends the concerns to raise important questions about inequities in retirement.

